# Investigating long-term trophic stability in North Atlantic cod (*Gadus morhua*) through nitrogen stable isotope analysis of amino acids

**DOI:** 10.1098/rstb.2024.0028

**Published:** 2025-07-10

**Authors:** Afrifa Kwaku Kyei Yamoah, Jennifer Harland, Rowan McLaughlin, Helen M. Talbot, Maria Fontanals-Coll, Oliver E. Craig, David Orton

**Affiliations:** ^1^ Department of Archaeology, University of York, York, UK; ^2^ Archaeology Institute, University of the Highlands and Islands Orkney College, Kirkwall, Orkney, UK; ^3^ Maynooth University, Maynooth, Ireland

**Keywords:** compound-specific isotope analysis of amino acids, trophic variability, North Sea, marine food web, Atlantic cod

## Abstract

Human-induced environmental change and fishing pressure have deleterious effects on marine ecosystems, but beyond that, the longer-term impacts are much harder to assess. Here, we applied bulk nitrogen isotopes (*δ*^15^N_Bulk_) and compound-specific isotope analysis of amino acids (*δ*^15^N_AA_) to well-dated cod remains from northeast Scotland to provide insights into the trophic structure in the North Sea over the last 1500 years. Ontogenetic changes were observable in trophic *δ*^15^N_AA_ and *δ*^15^N_trophic-source_ proxies but not in *δ*^15^N_Bulk_, questioning the latter’s use for inferring trophic level changes. We deployed a Bayesian generalized additive model, incorporating size-related uncertainties, to show that the trophic level of cod remained relatively stable from 500 CE to 1800 CE despite major climate and economic transitions. However, in the last 200 years, the *δ*^15^N_trophic-source_ proxy increased against the expectations of the effects of overfishing. While an increase in the trophic level of cod may be attributable to a restructuring of the North Sea food web owing to overfishing, other variables such as stress and diet quality might have affected nitrogen isotope fractionation, leading to similar outcomes. Our results show that multiple factors could drive *δ*^15^N through time; thus, physiological and biochemical factors must be considered when evaluating long-term trophic dynamics.

This article is part of the theme issue ‘Shifting seas: understanding deep-time human impacts on marine ecosystems’.

## 1. Introduction

Identifying human impact on marine ecosystems is a common goal within historical marine ecology. However, systematic scientific observations typically only stretch back a few decades and rarely over a century; even the earliest records may potentially represent systems already subject to significant decline [1,2]. Rather than proposing hypothetical ‘baselines’ in complex and inherently dynamic systems, it is perhaps better to think in terms of thresholds in the scale of anthropogenic drivers [3]—an approach that necessarily requires long-term datasets.

Archaeological evidence may be valuable in this context, typically extending beyond the scope of written records and often providing more-or-less continuous series over considerable time periods, albeit at lower temporal resolution than historical records. In general, zooarchaeological finds—i.e. physical remains of targeted species recovered from archaeological sites—represent direct evidence of past exploitation and indirect evidence of the populations and ecosystems involved [4]. Relative frequencies of taxa and skeletal elements can provide qualitative indications of shifts in scale, scope and methods of marine resource exploitation [5,6], but unlike some historical datasets cannot usually be used to estimate, e.g. biomass extractions or catch-per-unit-effort. Changes in catch-size distributions inferred from bone finds may likewise be an indirect indicator of trends in underlying stocks, but mediated through cultural, economic and technological factors [7–9].

On the other hand, individual zooarchaeological specimens do represent direct biological samples specifically of targeted populations. Stable isotope analysis (SIA)—a routine tool in contemporary trophic ecology—is now widely applied to collagen (the major bone protein) from archaeological fish remains in order to infer catch environments [10,11], trace changes in trophic webs [12,13], or even detect imports from distant water [14,15]. Stable nitrogen isotope values (*δ*^15^N) in particular are frequently used as an indicator of trophic level [16–18] with the potential to detect shifts over time owing to fishing pressure [19–21] or climatic drivers [22,23].

Impacts of overfishing on the trophic dynamics of an ecosystem can be complex and hard to predict, especially where multiple taxa are exploited at varying intensities (e.g. [19,24,25]). Since fishing typically removes the largest individuals, and size is a key determinant of diet in piscivorous taxa [26,27], mean trophic levels of heavily exploited species are expected to decline [18], although this may to some extent be compensated by reduced intra-specific competition and increased piscivory among smaller individuals [28]. Meanwhile, where fisheries also target mid-level forage fish such as herring, *δ*^15^N values of top predators are expected to decrease owing to reduced average trophic level of available prey—similar to the proposed phenomenon of fishing down the food web among human fisheries [29,30].

The potential of nitrogen isotope analysis on archaeological specimens to explore long-term trophic trends has been limited in practice by three main factors. First, *δ*^15^N at the base of marine food webs varies both spatially and temporally, confounding trophic effects in conventional studies of bulk collagen stable isotope analysis (bulk SIA). Compound-specific isotope analysis of amino acids (*δ*^15^N_AA_) has the potential to isolate trophic from baseline effects (e.g. [31–35]) but has yet to be widely applied to archaeological fish remains. Second, ontogenetic dietary trends in many fish species [36] require the size of sampled individuals to be taken into account systematically to explore changes in trophic-level-at-size. Third, the highly variable but typically low chronological resolution of archaeological data complicates the construction of robust time series [37].

Here, we combine analysis of *δ*^15^N_AA_ in bone collagen with Bayesian probabilistic modelling of both fish size and archaeological dating in order to assess trends in trophic level of Atlantic cod (*Gadus morhua*) over the past 1500 years in the northern North Sea. While the impact of recent industrial fishing on stocks of major food fish in this region is well-established [38,39], particularly after the introduction of steam-powered trawlers in the late nineteenth century [40], the possibility that pre-industrial—but nonetheless large-scale—demersal fisheries might already have impacted North Atlantic ecosystems during historical periods of increased fishing intensity, remains an untested hypothesis. Accordingly, we construct a time series of isotopic data spanning key developments in sea fishing in the region from its emergence as a large-scale practice up to and including recent industrialization. This study represents, to our knowledge, the first application of compound-specific isotope analysis to examine long-term changes in a historic fishery.

### (a) Background: northern Scottish fisheries

Some degree of reliance on marine resources appears to have been a constant in northern Scotland from early prehistory onwards, based on archaeological evidence, but an increase in coastal fishing is apparent during the Iron Age, approximately 700 BCE to 900 CE [41]. The arrival of the Vikings towards the end of the first millennium CE introduced a maritime-oriented culture and associated technologies to the region, including the first use of methods for intensive exploitation of marine resources [5]. Large gadoid species like cod, ling, saithe and pollack were particularly targeted [9,14], a development linked to the ‘Fish Event Horizon’: a wider increase in sea fishing, market demands and the use of preserved fish products around the North Sea region from approximately 1000 CE [5,42,43]. Fish remains are abundant on archaeological sites in northern Scotland in this period, reflecting both subsistence fishing and dumps of processing waste associated with proto-commercial fishing [9].

This period of intensive fishing started to decline in northern Scotland during the fourteenth and fifteenth centuries, probably owing to loss of regional markets for preserved fish, worsening climate making fishing in deeper waters difficult and unpredictable, and long-term societal effects of the fourteenth-century Black Death [44].

Exploitation of gadids and herring in the northern North Sea continued and intensified through the early modern period, however, largely in the form of distant-water fisheries—part of a wider fisheries expansion from approximately 1500 CE that has been termed the ‘North Atlantic fishing revolution’ [45]. The Northern Isles herring fishery was frequented by Dutch, French and English fishers between the fifteenth and seventeenth centuries; at times, the Dutch used hundreds of herring ‘busses’ to fish in the waters off Shetland and, to a lesser degree, Orkney. The wider pattern of fishing across the North Atlantic indicates cod catches increased through these centuries, exceeding herring landings [46], but this was not the case in the Northern Isles: historical sources show that fishing effort for herring greatly exceeded that of other species in this particular region [47]. There is some evidence for fishers from the Scottish east coast towns fishing for cod and ling in the sixteenth century [48], while the Dutch conducted some cod and ling fisheries year-round off of the Northern Isles during at least the seventeenth century [47]. German merchants operating out of coastal booths in Shetland purchased cod and ling from local fishers, salting and drying them using imported continental salt, and exporting them to continental markets in the sixteenth to early eighteenth centuries [49]. These commercial efforts have left little (zoo)archaeological trace, even at excavations of German booth sites in Shetland. Since little commercial-scale fishing was undertaken by the inhabitants, and since much of the fish biomass extracted by foreign fishers was taken to urban markets without ever being landed in the Northern Isles [46], archaeological data from these centuries is sparse. This has been exacerbated by limited archaeological interest in the early modern period, but recent excavations show that local subsistence-level fishing persisted, sometimes including large (upwards of approximately 700 mm total length; occasionally as high as approximately 1200 mm) cod in proportions that suggest deep water rather than close inshore fishing.

The fishing industry saw a further significant resurgence in the nineteenth century owing to technological advancements and capital investment at the tail end of the Industrial Revolution—notably the introduction of steam-powered boats and mechanised trawling gear—which improved fishing efficiency and turned it into a profitable industry. The expanded ability of marine exploitation, especially of herring and cod, resulting from industrialization led to overfishing by the early 1900s [46,50].

## 2. Material and methods

### (a) Study area and sampling of cod bone

**Figure 1.**
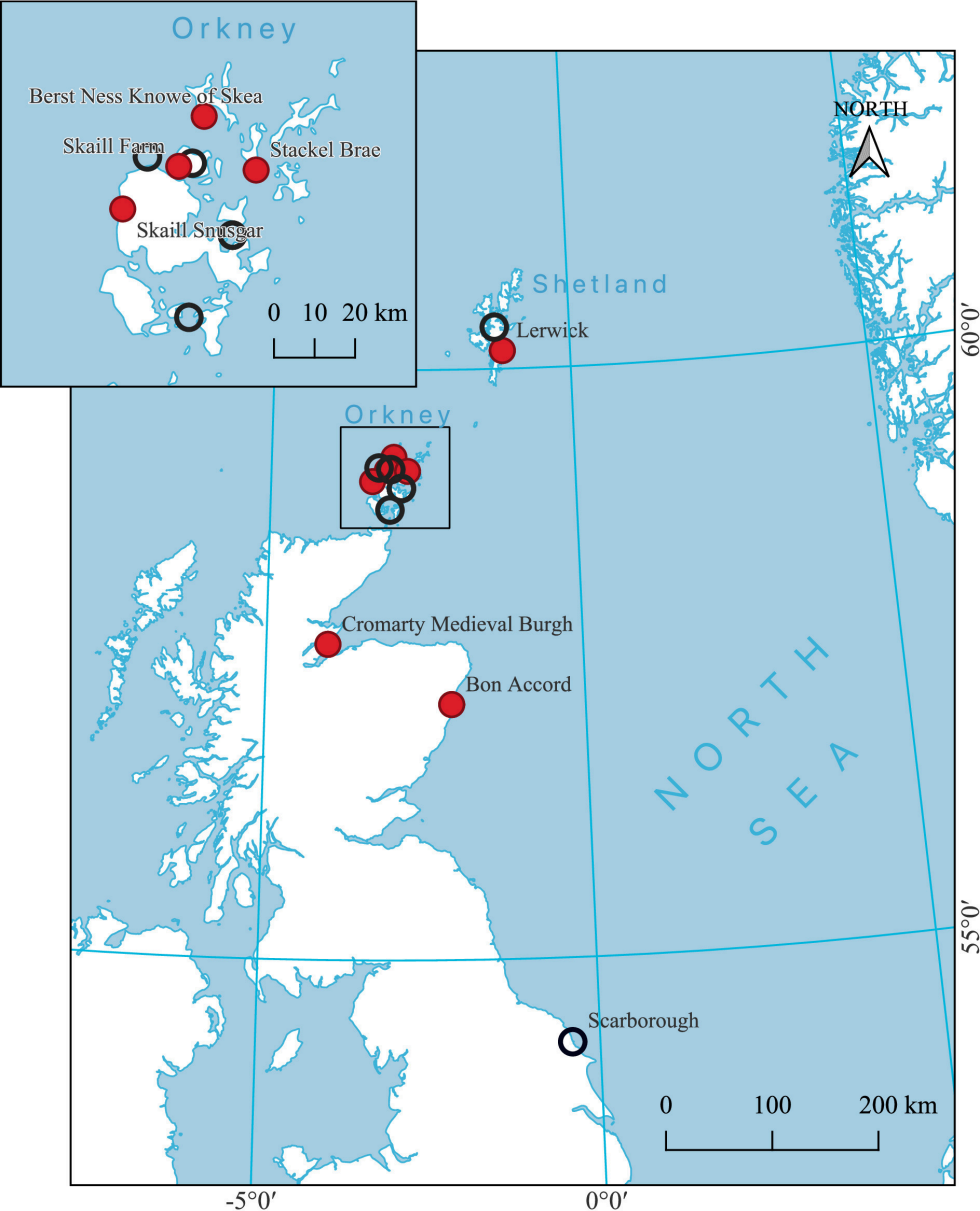
Map of the study area showing the location of archaeological sites (filled circles) and approximate modern locations (empty circles). Coastline from https://www.naturalearthdata.com/ and archaeological site locations from CANMORE (https://canmore.org.uk/).

A total of 150 cod bones were selected from the over 121 000 fish bones excavated at archaeological sites in northern Scotland, including specimens from Berst Ness Knowe of Skea, Bon Accord, Lerwick, Skaill Farm, Skaill Snusgar, Stackel Brae and Cromarty Medieval Burgh (figure 1). During excavation, stratigraphic sequences were carefully recorded so retrieved fish bones could be matched with typologically dateable cultural artefacts. Additionally, radiocarbon dates on terrestrial materials associated with contexts containing fish bones were obtained from 20 samples from Skaill Farm, Skaill Snusgar and Stackel Brae (SUERC Radiocarbon Laboratory). The radiocarbon dates confirmed the contextual dating and ensured a high degree of confidence in the chronological placement of the archaeological fish bones. Specimens for isotopic analysis were selected so as to avoid duplicate sampling of individual cod as best possible: within each stratigraphic context, multiple bones were only sampled where they were demonstrably derived from discrete individuals, either representing the same anatomical element and side or being of clearly different sizes.

In addition, 13 modern cod specimens—caught between 2010 and 2022—were sampled. Of these, six samples were caught off Orkney; one off Shetland and six were caught off the east coast of northern England and landed at Scarborough. Fish bones were treated with boiling water to facilitate the physical removal of soft tissues. The bones were dried at ambient room temperature and defatted using a 2 : 1 v/v solution of dichloromethane and methanol solution, rinsed three times and allowed to dry in a fume hood for 48 h.

### (b) Cod size estimation

Since trophic level of cod is expected to increase with size [51,52], it was important to estimate the size-at-capture of sampled individuals. This was done in two ways. First, all specimens were assigned a relative size class (e.g. ‘800−1000 mm’) based on comparison to modern reference specimens of known total length. Second, anatomical measurements were taken on all specimens whose state of preservation permitted it, following published protocols [53]. For cranial elements, specifically dentary, premaxilla and basioccipital, these were used to calculate an estimated total length (ETL) using bivariate and multivariate quadratic regression models built from equivalent measurements on 72 modern cod skeletons of known total length.

### (c) Collagen extraction

The selected archaeological fish bones were cleaned using a Vaniman Master Problast 3 sandblaster to eliminate potential post-depositional surface contamination. Before proceeding to the collagen extraction, the samples (approx. 100−200 mg), both modern and archaeological, underwent a defatting procedure by sonicating them for 15 min in a 2 : 1 dichloromethane/methanol solution (v/v). This process was repeated at least three times, ensuring that the solvent mixture became clear of any lipids before proceeding. Afterwards, any remaining solvent was allowed to evaporate overnight. To ensure complete removal of residual solvents, the samples were rinsed with deionized water three times.

Both the selected cod bones and modern bovine bone controls were demineralized using 8 ml of 0.6 HCl at 4°C for 2−4 days for the archaeological cod samples and 7−10 days for the modern cod samples, followed by ultrapure water rinsing (milli-Q®) and gelatinization with 0.001 M HCl at 80°C over 48 h. Subsequently, polyethylene Ezee filters (60−90 μm, 9 ml) were used to filter the samples before they were frozen for 24−48 h at −20°C. The samples were then freeze-dried, and weighed (0.3−0.5 mg) into tin capsules to be measured in the elemental analyser—isotope ratio mass spectrometer (EA-IRMS) at the BioArCh laboratories, located within the Department of Archaeology at the University of York, UK.

### (d) Bulk isotope analysis by elemental analyser—isotope ratio mass spectrometer

The collagen samples were analysed in duplicate on a Sercon continuous flow 20−22 IRMS combined with a Universal Sercon gas–solid–liquid EA. The carbon (*δ*^13^C) and nitrogen (*δ*^15^N) isotopes values were corrected for Vienna Pee Dee Belemnite (VPDB) and air (AIR), respectively, and were reported in *δ* (‰) notation. International standard reference materials were run together with the samples in order to ensure accurate isotopic values. The *δ*¹³C values were: Iso-Analytical (IA) cane, −11.59 ± 0.03‰ (raw) and −11.64‰ (true); methionine, −35.61 ± 0.09‰ (raw) and −35.83‰ (true); fish gel, −15.13 ± 0.09‰ (raw) and −15.27‰ (true); alanine, −23.14 ± 0.01‰ (raw) and −23.33‰ (true); and IA soy, −25.02 ± 0.01‰ (raw) and −25.22‰ (true). For *δ*¹⁵N values: International Atomic Energy Agency (IAEA) N2 standard, 20.40 ± 0.12‰ (raw) and 20.41 ‰ (true); methionine, −1.13 ± 0.16‰ (raw) and −0.76‰ (true); fish gel, 15.36 ± 0.07‰ (raw) and 15.21‰ (true); alanine, −5.81 ± 0.04‰ (raw) and −5.56‰ (true); and IA soy, 0.80 ± 0.02‰ (raw) and 0.99‰ (true). Uncertainties were obtained by propagating those from reference materials and those from repeated sample measurements following [54]. The maximum uncertainty across all samples and runs remained below 0.2‰ for *δ*^13^C and *δ*^15^N, ensuring high precision and reliability throughout the analysis. Additionally, a bovine bone control in the same batch produced *δ*^13^C = −23.16 ± 0.23‰ and *δ*^15^N = 6.81 ± 0.34‰, consistent with an average from 50 separate extractions, showing *δ*^13^C = −23.26 ± 0.14‰ and *δ*^15^N = 6.39 ± 0.51‰.

### (e) Sample preparation and analysis of amino acids by gas chromatography-combustion-isotope ratio mass spectrometry

The sample preparation for SIA followed the procedure described in Soncin *et al.* [55]. Briefly, about 4 mg of collagen was hydrolysed into its constituent amino acids (AAs) by adding 6 M HCl (200 μl) and heating at 110°C for 24 h. Prior to this, an internal l-norleucine standard (Sigma-Aldrich; 50 μl equivalent to 25 μg) with known isotopic composition (13.96‰) was added to each sample. This was adjusted for samples that were less than 4 mg. After hydrolysis, the samples were filtered using nanosep (0.45 μm) to remove insoluble matter from the hydrolysates before derivatization. During derivatization [56], the samples were esterified by adding isopropanol and acetyl chloride (1 ml; 4 : 1 v/v) and heated at 100°C for 1 h. This derivatized the AAs into their *i*-propyl esters. Subsequently, the AAs were acetylated to form *N*-acetyl-*i*-propyl derivatives using acetic anhydride, triethylamine and acetone (1 ml; 1 : 2 : 5, v/v/v) and heated at 60°C for 10 min. Samples were dried under nitrogen.

One millilitre of saturated NaCl solution and 2 ml of ethyl acetate were added to the samples and vortexed for 20 s. The sample was divided into organic and aqueous phases, and the organic phase was removed into a new labelled vial. Ethyl acetate (1 ml) was added to the previous tube containing the NaCl, and the process was repeated. To eliminate trace water, sodium aluminium silicate molecular sieves (0.3 nm) were added to the supernatants in the newly labelled vial. The ethyl acetate was transferred to a gas chromatography (GC) vial, dried under N2 and then diluted for onward GC-combustion-IRMS (GC-C-IRMS) analysis. International reference standards (from Indiana, USA and SHOKO Science, Japan) were also prepared using the same procedure.

The isotopic analysis of the AA was carried out on a paired Delta V Plus IRMS and Trace Ultra 1310 gas chromatograph (Thermo Fisher Scientific in Bremen, Germany) equipped with a GC IsoLink II interface containing a Cu/Ni combustion reactor operating at 1000°C. Ultra-high purity helium was used as the carrier gas at a flow rate of 1.4 ml min^−1^. The column used for the analysis was a custom DB−35 fused silica column (60 m × 0.32 mm × 0.50 µm; Agilent J&W Scientific Technologies, Folsom, CA, USA) at 240°C with the injection volume for samples and standards being 1 and 2 µl, respectively. The oven temperature programme consisted of holding at 40°C for 5 min, increasing by 15°C min^−1^ to 120°C, then by 3°C min^−1^ to 180°C, then by 1.5°C min^−1^ to 210°C, and finally by 5°C min^−1^ to 280°C, holding for 8 min. All samples were injected two or three times and analysed for 15N : 14N ratio using ion intensities of *m/z* 28 and 29 in nitrogen mode. A Nafion membrane and cryogenic trap was used to remove water and CO_2_, respectively, during the analysis. Results from the analysis were initially measured using Isodat (v. 3.0; Thermo Fisher Scientific) before the LyticOS software by Elementar was used to compute results based on repeatedly measured high-purity AA standards. Results are presented in parts per mil (‰) relative to international standards using the δ notation.

### (f) *δ*^15^N measurements of amino acids and quality control criteria

To determine the reported *δ*^15^N values, duplicate and triplicate measurements were taken, and instrument performance and drift were monitored using a mixture of international reference standards of known isotopic ratio after every three duplicated sample injections. The AA standard mixture included eight international standards (Indiana and SHOKO Science) and l-norleucine (Sigma-Aldrich). The samples were analysed in multiple batches, with each batch calibrated using its own specific calibration curve in order to ensure that each set of samples were calibrated according to the conditions under which they were analysed. The average raw values and s.d. (*n* = 289) for international standards were: alanine (Ala) 41.50 ± 2.33‰ (true: +43.25 ± 0.07‰), glycine (Gly) −1.08 ± 1.44‰ (true: +1.76 ± 0.06‰), valine (Val) −6.68 ± 1.84‰ (true: −5.21 ± 0.05‰), leucine (Leu) +4.4 ± 1.20‰ (true: +6.22‰), norleucine (Nle) +12.60 ± 1.26‰ (true: +13.96 ± 0.23‰), aspartic acid (Asx) +32.99 ± 1.54‰ (true: 35.2‰), glutamic acid (Glx) −5.53 ± 0.76‰ (true: −4.52 ± 0.06‰), hydroxyproline (Hyp) −9.95 ± 0.70‰ (true: −9.17‰) and phenylalanine (Phe) +0.70 ± 0.64‰ (true: +1.70 ± 0.06‰). The sample *δ*^15^N raw values were corrected using the calibration curve.

Using GC-C-IRMS AA measurements, we captured 80.7% of fish collagen’s total nitrogen. We calculated *δ*^15^N values for collagen using mass balance equations that considered AA contributions. We compared with EA-IRMS results, excluding samples exceeding 2*σ* observed offset. The mean Δ^15^N_Est-Obs_ across all samples was 0.22‰ ( ± 0.81‰). Additionally, we tracked the correlation between stable isotope values of proline and hydroxyproline owing to biosynthetic similarities.

### (g) Statistical analysis

Principal component analysis (PCA) was used to characterize the covariability in the isotopic data, in particular, to determine that metabolic differences between trophic and baseline AAs are captured by the PCA factor loadings. Linear relationships between individual AAs were quantified using a correlation coefficient (Pearson’s product moment correlation coefficient, *r*) expressing the strength and direction of linear associations. A formal test of association yielded *p*-values used to confirm the statistical significance of the results, ensuring that the relationships observed were not owing to random variation. We also tested the relationship between the bulk *δ*^15^N results, estimated fish length, the AAs and the baseline-adjusted trophic signal (Δ^15^N_Glx-Phe_) results in this way. This analysis was conducted in *R* v4.1 and the default *stats* package, with the *corrplot* library [57] used for visualization.

### (h) Generalized additive models

We fitted our data to generalized additive models (GAMs) to produce time series models of changes to trophic and source AAs while simultaneously modelling the effect of fish size on the trophic position (TP) of each organism. The basic model is:


(2.1) Y = s(t) +x + ϵ,

where *Y* is the measured *δ*^15^N_AA_ or Δ^15^N_AA-AA_ value, *s*(*t*) is a smooth function of time, *x* is an estimate of fish total length and ϵ is an error term. Analytical complexity is introduced by machine measurement error on *Y*, and significant uncertainty in estimating the chronological age *t* and size *x* of these archaeological fish samples. Chronological age estimates are based upon an assessment of archaeological and historical data, resulting in a uniformly distributed estimate for each sample bounded by *t*_0_ and *t*_1_. Fish size estimates were made as discussed in §2.2 above and their uncertainty modelled using either regression results or through fitting a normal distribution to relative size classes. We used Bayesian inference to estimate the parameters of the smoothing function *s* given the uncertainty of *Y* and *x*, with the model (2.1) specified as:


(2.2) Y = s(t)+ Z +η+ ϵ,

where η is machine uncertainty and *Z* is a probability distribution where $Z\;∼\;normal(\underset{\_}{x},\sigma^{2})$ and $t\;∼\;uniform\left(t_{0},t_{1}\right)$. It was not computationally feasible to estimate the posterior probability distribution for *t* while also fitting the smoothing terms *s* (i.e. estimating the dimension and hyperparameters of the basis functions used for the smoothing spline), so we developed two pragmatic solutions to address the problem of age uncertainty. The first was to run the model (2.2) multiple times using different point estimates of *t* and compare the conditional effects of the model predictors, to check that age uncertainty was not biassing the results. Second, for subsets of the data where a strongly linear trend was apparent in the GAM, we re-specified the model (2.1) using linear regression


(2.3) $$Y\;∼\;normal\left(\eta \;+\alpha +\beta_{0}\;t\;+\beta_{1}x\;,\sigma^{2}\;\right),$$

where α and β are intercept and slope, and used Bayesian inference to estimate the full posterior distributions of all model parameters in equation (2.3). We also modified equation (2.2) to generate a continuous estimate of TP [34]. This was done in the Bayesian model by transforming *Y* (in this case the Δ^15^N_Glx-Phe_ measurement) according to:


(2.4) $$TP\;=\left(Y-2.9\right)\;/\;TEF\;+1_{,}\;,\;TEF\;∼\;normal\left(6.6,\;1.7\right),$$

where TEF is a trophic enrichment factor. The 2.9 in equation (2.4) is the primary producer AA_tropic-source_ offset which was set, along with the choice of prior for TEF, 6.6 ± 1.7, following discussion in Nielsen *et al.* [32]. We used the *R* packages *brms* [58] and *mgcv* [59] to set up the *Stan* (Stan Development Team 2024) models that performed this analysis. Computer codes to replicate all the analysis and all the datasets are archived at the Zenodo digital repository (doi: 10.5281/zenodo.14016873).

## 3. Results

### (a) Quality control

The suitability of the collagen used for the CSIA was assessed using similar parameters described in Soncin *et al.* [55]. A total of 13 proteinogenic AAs comprising trophic AAs, which included Glx, Ala, Asx, proline (Pro), Leu and Val and source AAs, which were Phe, Gly, serine (Ser) and lysine (Lys) were measured. Other AAs which have been described as metabolic AAs, which were also measured included isoleucine (Ile), threonine (Thr) and Hyp.

The initial quality control was based on the atomic C : N ratio of bulk collagen. Ratios between 3.0 and 3.6 were considered acceptable [60], while any outside this range were discarded. Based on the *δ*^15^N measurements of the individual AAs and their percentage contributions to the bulk collagen, the bulk nitrogen value was estimated (*δ*^15^N_Est_). The discrepancy between estimated (*δ*^15^N_Est_) and observed (*δ*^15^N_Obs_) values was assessed to evaluate the accuracy of our estimates. About 65% of the *δ*^15^N_Est-Obs_ differences were less than ±1.0‰, indicating close agreement between the estimated and observed values. The remaining 35% of values ranged from 1.0‰ to 3.5‰, with most of these clustering towards the lower end of the range. This discrepancy may be attributed to the absence of an ultra-purification step in our collagen extraction process, where non-collagenous proteins and other impurities are potentially retained in the collagen matrix. Equally, discrepancies in the *δ*^15^N_Est_–*δ*^15^N_Obs_ may reflect the fact that we only measure approximately 80% of the nitrogen in collagen through CSIA.

**Figure 2.**
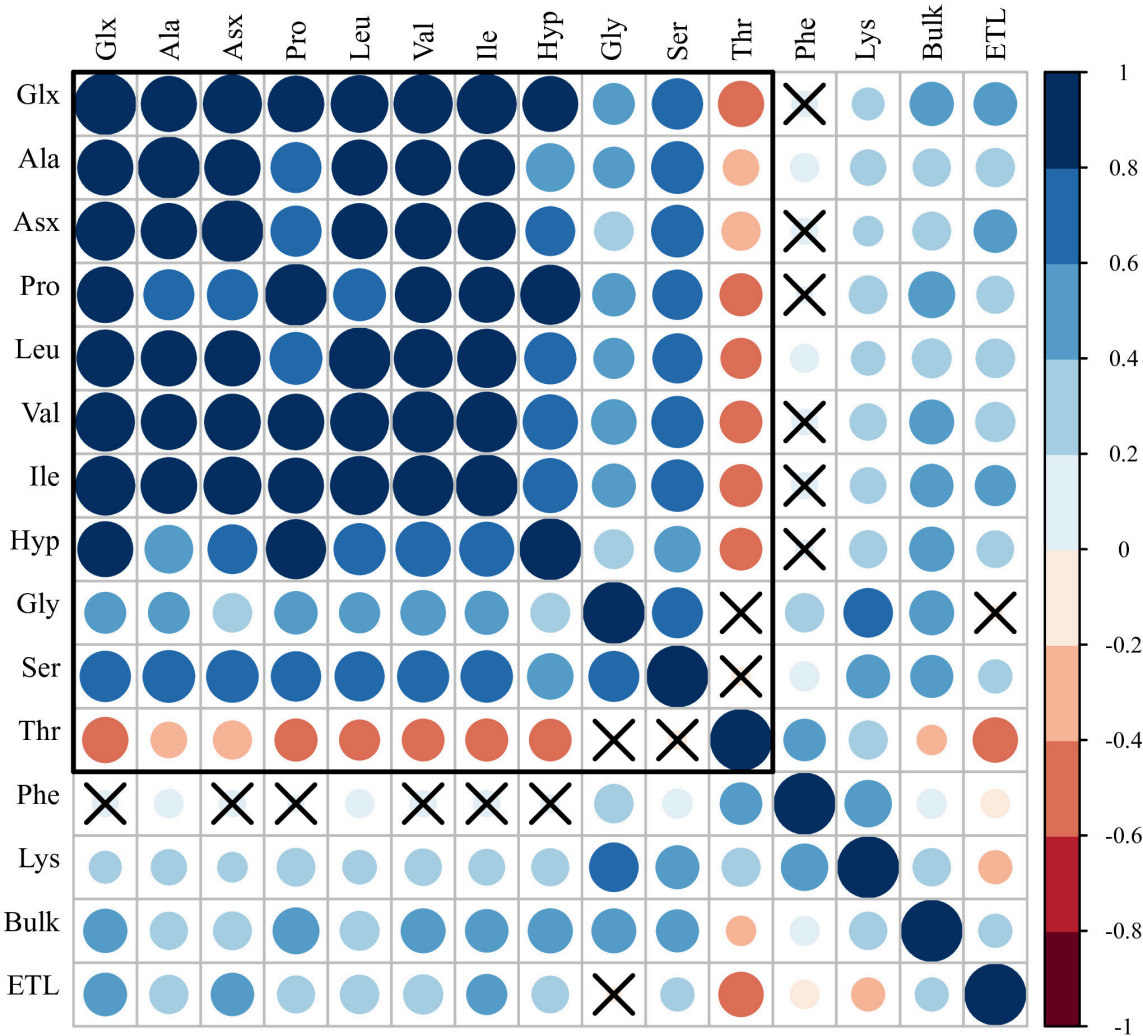
Correlation diagram (Pearson’s product moment correlation coefficient) between pairs of *δ*^15^N of individual amino acids and *δ*^15^N_Coll_ (bulk) and the estimated fish length (ETL). Cases where the test of association is not significant (i.e. two-sided *p* = 0 > 0.05) are indicated with a cross. Trophic and metabolic amino acids are grouped within the frame encompassing the 11 dimensions on the upper left.

That notwithstanding, the Hyp, which differs from Pro by having a hydroxyl (OH) group attached to the gamma carbon atom, exhibited minimal variation in isotopic composition, showing a strong correlation (*r* = 0.88). This consistency highlights the robustness of the analytical method (figure 2; electronic supplementary material, table S1).

### (b) Bulk and compound-specific isotope analysis of amino acids of cod collagen

Differences in *δ*^15^N between individual AAs are expressed using PCA. The first two dimensions of the principal component (PC) scores (PC1 and PC2) accounted for 84% of the total variance observed in the dataset. PC1 is strongly loaded with trophic AAs, with various degrees of loading from source AAs. This indicates a substantial positive contribution to the variance explained by this PC. Thr, which is the only AA that exhibited high negative loading on PC1 typically exhibits a unique trend of decreasing *δ*^15^N values with increasing trophic levels as a result of preferential enzymatic breakdown of ^15^N-threonine into ammonia and alpha-ketobutyrate leading to a reduction in ^15^N relative to the dietary source [61]. Predictably, the source AAs Lys and Phe had very weak loadings against PC1.

The correlation matrix exploring the relationships between trophic AAs (figure 2; see also the electronic supplementary material, table S1) shows strong correlations among individual trophic AAs. *δ*^15^N_Glx_, which is the most widely used AA for estimating trophic level demonstrates a robust correlation with other trophic AAs *δ*^15^N_Ala_ (*r* = 0.812), *δ*^15^N_Val_ (*r* = 0.906), *δ*^15^N_Leu_ (*r* = 0.854), *δ*^15^N_Ile_ (*r* = 0.931), *δ*^15^N_Pro_ (*r* = 0.822) and *δ*^15^N_Asx_ (*r* = 0.902). These AAs either readily interchange their α-carbon through transamination reactions, primarily with glutamic acid (Glu), or derive their nitrogen from the same metabolic pool during biosynthesis [62]. Thus, any of these AAs could be an effective proxy for trophic level estimates, as suggested in some studies [32–34,63]. However, this has often led to overestimations and inaccuracies [64]. Glu, represetned here as *δ*^15^N_Glx_, is considered a more reliable trophic indicator owing to its central role in key metabolic processes compared to other trophic AAs [62].

As expected, the ‘metabolic' AA Thr shows a strong negative correlation with the other trophic AAs owing to its serial depletion with trophic level [61]. Gly is reasonably correlated with Ser owing to their close metabolic relationship and bulk collagen where it is a major contributor (*ca* 30%) to the total nitrogen. Gly and Ser acids are sometimes considered as source AAs but can also undergo extensive transamination with the nitrogen pool and with ammonia during urea production [63]. In this system, they were not strongly correlated with the other trophic AAs and behaved more like source AAs (see figure 2).

**Figure 3.**
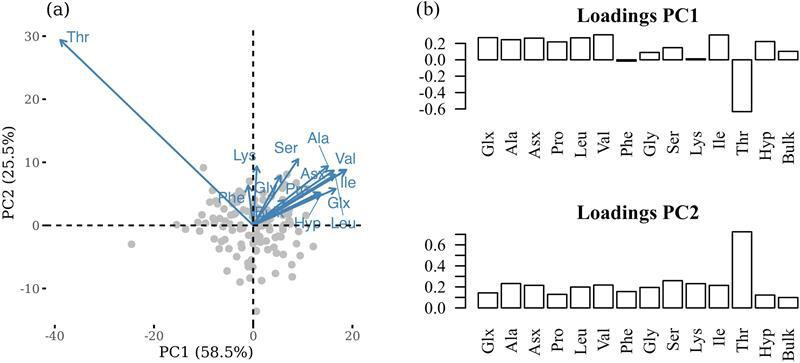
PCA plots (*a*) biplots of isotopes of individual amino acids and bulk collagen, and (*b*) factor loadings of *δ*^15^N of individual amino acids and including ‘bulk’ *δ*^15^N_Coll_.

Of the other source AAs, *δ*^15^N_Phe_ and *δ*^15^N_Lys_ show the lowest correlations with *δ*^15^N_Glx_ (*r* = 0.156, *p*‐value = 0.087 for *δ*^15^N_Phe_ and *r* = 0.249, *p*‐value = 0.006 for *δ*^15^N_Lys_), suggesting minimal metabolic alteration. The clustering of *δ*^15^N_Phe_ and *δ*^15^N_Lys_ show an affinity between these two AAs (figure 3). However, the strong correlation between *δ*^15^N_Lys_ and *δ*^15^N_Gly_ probably suggest some degree of metabolic alteration compared with *δ*^15^N_Phe_, which shows no correlation with either *δ*^15^N_Gly_ or *δ*^15^N_Ser_. We therefore consider *δ*^15^N_Phe_ to be the most reliable source proxy. To effectively isolate the trophic signal from the baseline, we determine the TP (Δ^15^N_Glx-Phe_) by calculating the difference between *δ*^15^N_trophic_ (*δ*^15^N_Glx_) and *δ*^15^N_source_ (*δ*^15^N_Phe_).

The ETL of the fish, shows only a weak correlation with *δ*^15^N_Coll_ (*r* = 0.27, *p*‐value = 0.0023). Interestingly, *δ*^15^N_Gly_, the most abundant AA in collagen, shows no correlation with ETL (*r* = −0.02, *p*‐value = 0.843) confirming its behaviour as a source AA. Similarly, the correlation between ETL of cod and the isotopic signatures of other source AAs, *δ*^15^N_Phe_ (*r* = −0.13, *p*‐value = 0.14) and *δ*^15^N_Lys_ (*r* = −0.21, *p*‐value = 0.02), were found to be weak and/or insignificant and therefore most appropriate (especially for *δ*^15^N_Phe_) for use as proxy for the *δ*^15^N of organisms at the base of the food web. By contrast, we observe low to moderate correlations between ETL and the trophic AAs, *δ*^15^N_Glx_ (*r* = 0.482), *δ*^15^N_Ala_ (*r* = 0.380), *δ*^15^N_Val_ (*r* = 0.405), *δ*^15^N_Leu_ (*r* = 0.389), *δ*^15^N_Pro_ (*r* = 0.374) and *δ*^15^N_Asx_ (*r* = 0.473). The correlation strengthens when considering the baseline-adjusted trophic signal, Δ^15^N_trophic-source_ (Δ^15^N_Glx-Phe_ *r* = 0.503).

### (c) Trophic and source amino acid variability over the last 1500 years

**Figure 4.**
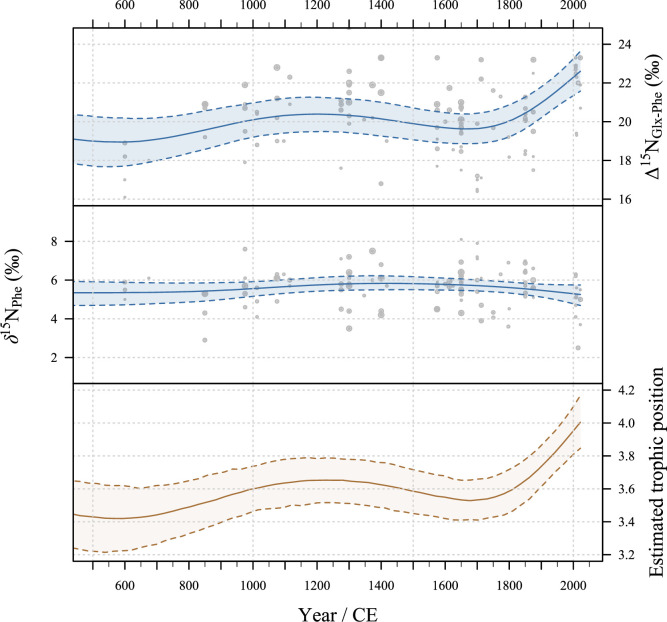
A time series for Δ^15^N_Glx-Phe_ and *δ*^15^N_Phe_ and a predicted trend based on GAM Bayesian model output, incorporating the cod length and age uncertainties. Each circle represents an isotope ratio measurement, with the radius proportional to estimated total length. Also indicated is the changing estimated trophic position of cod while holding size constant, based on the equation of Chikaraishi *et al.* [34] and using trophic enrichment factors suggested by Nielsen *et al.* [32].

We generated GAM models for all the key trophic indicators—Δ^15^N_trophic-source_, *δ*^15^N_coll_ and *δ*^15^N_Phe_ as well as Δ^15^N_Glx-Phe_, Δ^15^N_Glx-Lys_, Δ^15^N_Pro-Phe_, Δ^15^N_Pro-Lys_ and *δ*^15^N_Lys_ to investigate changes in these offsets over time (see figure 4 and the electronic supplementary material, figure S5). We also generated GAM models for estimated TP of cod (figure 4) using the equation and TEF suggested by Chikaraishi *et al.* [34] and Nielsen *et al.* [32], respectively. While some samples date to the first millennium BCE, the sparseness of these data limits our capacity to detect any diachronic trend. We therefore concentrate our discussion on the last 1500 years, where our higher sampling resolution permits us to evaluate the significance of changes we observe in our data. After accounting for the uncertainties associated with the ages and sizes of the cod bones, and the interaction between these variables, we observe some oscillations in the Δ^15^N_Glx-Phe_, *δ*^15^N_Phe_ and estimated TP modelled trends (figure 4). However, until approximately 1800 CE these fluctuations fall within the margin of error in the GAM predictions, implying that any pre-industrial changes in trophic ecology and the nitrogen baseline remain undetectable using this method (see the electronic supplementary material, figure S6). Significantly, however, Δ^15^N_Glx-Phe_ and estimated trophic level rose markedly between 1800 CE and the present day, although we cannot at present be sure whether this increase began during the eighteenth, nineteenth or twentieth centuries. *δ*^15^N_Phe_ followed a slight downward trajectory over the same period (figure 4). The strength of the former trend is such that our Bayesian model is able to calculate posterior probability distributions for the date of the fish that improve upon the original age estimates for historic specimens (see the electronic supplementary material, figures S2 and S3), and it remains present albeit with reduced confidence when 13 recent specimens from Scarborough are removed from the sample (see the electronic supplementary material, figure S6).

## 4. Discussion

### (a) Size-dependent variations in *δ*^15^N of bulk collagen and amino acids of cod

The relationship between the size of cod, as determined by their ETL, and the isotopic composition of bulk collagen and individual AAs was investigated. The low-to-moderate correlations between the ETL of cod and the *δ*^15^N values of trophic AAs, except for *δ*^15^N_Gly_ (figure 2), indicate an ontogenetic dietary shift. As cod mature, they exhibit a dietary shift towards consuming prey at increasingly higher trophic levels, with juvenile cod preying on smaller fishes, which are more proportional to their body length while larger cod consume prey of varying sizes [65]. These dietary changes contribute to the measurable enrichment in *δ*^15^N values of trophic AAs as the cod increases in size (figure 2; electronic supplementary material, figure S4). It is important to note that the use of bone samples, unavoidable with archaeological material, rather than a faster-turnover tissue such as skin or muscle will have resulted in some degree of time averaging over sampled individuals’ lifetimes, reducing apparent correlations between fish size and *δ*^15^N values [66]. True relationships between size and trophic level are thus likely to be stronger than indicated by our results, but it is not possible to quantify this effect with our current limited understanding of bone collagen turnover rates in fishes, which is likely to be influenced by growth rates, dietary quality and may vary considerably between different skeletal elements [66]. That said, we observed similar correlations between ETL and isotope results for the frequently sampled elements in our data (electronic supplementary material, table S2).

Interestingly, we observed a very weak correlation between ETL and *δ*^15^N_Coll_ (*r* = 0.27; figure 2). This was an unexpected and potentially highly significant finding given that *δ*^15^N_Coll_ is often used to estimate the TP and, therefore, should reflect ontogenetic variation in the size of carnivorous fishes [36]. For example, in modern natural populations of Norwegian cod, *δ*^15^N values of muscle tissue were found to be correlated with fork length, indicating that they consume at increasingly higher trophic levels as they increase in size [67]. For the historical bone specimens, there are numerous explanations as to why this may not be the case, including changes in baseline *δ*^15^N values through time, the time-averaging effect of sampling bone rather than muscle, or potentially differences in the AA composition between collagen and muscle proteins. Consideration of *δ*^15^N values of the AAs in bone collagen provides a means to investigate these further.

Indeed, we do observe correlations between the ETL and the trophic AAs reflecting ontogenetic variation in the size of cod. However, *δ*^15^N_Gly_ (comprising about 30% of collagen’s AA composition), shows no correlation with ETL (*r* = −0.02, *p*‐value = 0.843), similar to the correlations observed between the ETL and source AAs—*δ*^15^N_Phe_ (*r* = −0.13, *p*‐value = 0.14), and *δ*^15^N_Lys_ (*r* = −0.21, *p*‐value = 0.02)—which do not enrich trophic level. Gly, often considered a source AA, is primarily synthesized from the AA Ser through the enzyme serine hydroxymethyltransferase [68]. Although Gly can also be produced from Thr via threonine aldolase [69] and from choline conversion to betaine by betaine aldehyde dehydrogenase and choline dehydrogenase [70], the Ser pathway is most common. Therefore, it is not surprising that *δ*^15^N_Ser_ also shows a weak correlation with ETL similar to that of *δ*^15^N_Gly_ (figure 2). Indeed, in the collagen from the skin of beluga whales, *δ*^15^N_Gly_ was observed to be highly variable and not appropriate as a source AA, perhaps owing to some degree of synthesis as a result of the high metabolic demand of moulting [71]. With *δ*^15^N_Coll_ showing a significant correlation with *δ*^15^N_Gly_ (figure 2) and Gly being a significant component of cod collagen would explain why *δ*^15^N_Coll_ exhibits a weaker relationship with the ETL. Thus, *δ*^15^N_Coll_ lacks the precision and specificity required for detailed isotopic interpretations on trophic estimations. Bulk *δ*^15^N of different tissues with lower Gly content, such as muscle, feather or hair, might be more appropriate for estimating trophic level but these are rarely available archaeologically.

### (b) Trophic stability of cod from approximately 500 CE to approximately 1800 CE

In assessing the underlying factors—such as natural climate variability and human activities, including fishing pressure—driving the trophic variability of cod from approximately 500 CE to approximately 1800 CE, we compared the long-term isotopic patterns of Δ^15^N_Glx-Phe_ and *δ*^15^N_Phe_ (figure 2) with known historical trends in North Sea fishing and with known climatic transitions. Variability in Δ^15^N_Glx-Phe_ over time may suggest trophic dynamics [72] or may be a physiological response of cod in the marine food web [62,73]. By contrast, deviations in *δ*^15^N_Phe_ could point to changes in the phytoplankton primary production particularly in relation to changes in the N cycle, such as denitrification [74].

Our data shows that between approximately 500 CE and 1800 CE, changes in Δ^15^N_Glx-Phe_ and *δ*^15^N_Phe_ fall within the uncertainties associated with the analysis, including individual error propagation related to size and age uncertainties (figure 4). Interestingly, there were no observable impacts on these measures over major climatic transitions. Cooler temperatures from around 500 CE to 800 CE and from 1300 CE to 1700 CE during the Little Ice Age (LIA) and the intervening warmer period of the Medieval Climate Anomaly (MCA) appear not to have had any significant net impact on the trophic level of cod in the northeastern part of the North Sea nor the *δ*^15^N values of primary producers.

The relative stability of the trophic level of cod during this 500 CE–1800 CE period is also notable in the context of the major developments in human fishing activity outlined above for the study region. These include the approximately ninth century CE onset of large-scale marine fishing, the subsequent boom in gadoid fisheries through to the mid−fourteenth century, and the later emergence and intensification of long-range fisheries in northern Scottish waters—for cod and especially for herring, a key prey species for cod and other gadoids.

Our results do not show the decreasing trend in Δ^15^N_Glx-Phe_ that might be expected owing to ‘fishing down’ the foodweb—given the position of cod as an apex predator—if pre-industrial fishing activities had significantly impacted the marine ecosystem. Rather, we observe very little change over this period: Δ^15^N_Glx-Phe_ actually marginally increases during the medieval fishing boom, then marginally decreases during the early modern period, but both trends fall within the uncertainties of the analysis and individual error propagation.

### (c) Trophic instability of cod during the industrial period?

At some point after 1800 CE, the isotopic indicators start to diverge, with *δ*^15^N_Phe_ slightly declining while Δ^15^N_Glx-Phe_ begins to rise sharply (figure 4). This result is robust when we consider temporal uncertainty (see the electronic supplementary material, figure S1) and the potential effect of sampling location for the modern cod (see the electronic supplementary material, figure S6). This period broadly coincides with the onset of the Industrial Revolution, which was characterized by rapid growth in industrial activities, urbanization and agricultural practices. This rapid industrial growth could contribute significantly to the nutrient load in the North Sea, raising the likelihood of hypoxic events and eutrophication [75]). Interestingly, an increase in *δ*^15^N_Phe_ is not observed during this period as might have been expected owing to the effects of eutrophication on primary producers [76], perhaps indicating that the cod were feeding in waters peripheral to the main areas of North Sea eutrophication.

Overfishing during this period might also have had a significant impact on the trophic ecology of cod [77] the introduction of steam-powered trawlers in the late nineteenth century and the development of efficient fishing gear and methods during this period supporting large-scale fishing operations [39]. We do not, however, see the trend of declining Δ^15^N_Glx-Phe_—either in absolute terms or adjusted for ETL—that might be expected owing to fishing pressure on cod and their key forage fish herring. Instead, we observe a rising trend in size-adjusted Δ^15^N_Glx-Phe_ during this period (figure 4), suggesting that more complex trophic interactions and/or other factors may be driving the trophic variability of cod. This result contrasts with a recent study of modern and archaeological cod from eastern Iceland, in which a significant decline in *δ*^15^N of bulk bone collagen was observed from the nineteenth century, again coinciding with the onset of industrialized fishing [18].

It is important to note that our modelled increasing trend in Δ^15^N_Glx-Phe_ relates to trophic-level-at-size: we see fish of smaller average size apparently feeding at similar trophic levels to those previously seen in larger individuals. Reduction in cod stocks owing to overfishing may have relaxed intra-specific competition and increased availability of prey species, allowing a rise in trophic-level-at-size or even as the size of the average cod caught by humans decreased. Alternatively or additionally, overfishing of herring might have benefited other mid-level forage fishes. If species that are normally secondary or opportunistic prey for cod experienced a population increase this may have further supported the cod’s ability to maintain or even elevate their trophic level. The rise in Δ^15^N_Glx-Phe_ could thus be explained by ecosystem-wide restructuring, where mid-level species thrive under reduced predation pressure from overfished cod populations, allowing cod to access a more diverse or abundant prey base.

A second scenario is concerned with the TEF used to assess trophic level from the isotope measurements. Cod and their prey are likely to experience oxidative stress and poor physiological conditions as a result of both environmental degradation and overfishing. There is evidence to suggest that such deteriorating conditions could lead to an increase in TEF, i.e. the degree to which the *δ*^15^N values increase with each trophic level [62,73], which would increase Δ^15^N_Glx-Phe_ regardless of their TP. This may be partly attributable to slower growth rates [78] leading to enhanced catabolic processes and preferential excretion of isotopically lighter compounds [63] as well as greater isotopic routing of trophic AAs directly to collagen from the diet as a result of poorer or changing diet quality [79]. If the TEF values vary through time then the trophic level estimates presented in figure 4c will be included. Changing physiological conditions as a result of external environmental stress or changing diet quality present an additional confounding variable to consider when estimating long-term changes in trophic level.

## 5. Conclusion

The results of this study suggest that the application of bulk collagen *δ*^15^N as a trophic indicator for fish may be limited owing to its unusually high Gly content, which behaves like a source AA with a negligible TEF. However, considerable enrichment was apparent in trophic AAs with increasing estimated total length of cod, emphasizing the need to account for size-related uncertainties before deciphering any long-term patterns in the nitrogen isotope values.

Using a Bayesian GAM model that accounts for the size-related uncertainties, we show that the trophic level of cod remained relatively stable from 500 CE to 1800 CE despite significant climate variability (MCA and LIA) and major economic transitions resulting in increased fishing pressure: the Viking period fishing boom from approximately 900 CE , the ‘Fish Event Horizon’ approximately 1000 CE [5,80] and the expansion of longer-range fisheries linked to the wider ‘North Atlantic Fish Revolution’ approximately 1500 CE [45]. Although well documented and observable from the archaeological and historical records, respectively, we tentatively conclude that the latter were unlikely to have been of sufficient intensity to impact greatly on the mean trophic level of cod.

Subsequently, we observe an increase in Δ^15^N_Glx-Phe_ beginning at some point after 1800 CE, evident in our modern samples. We propose two possible interpretations: (i) increased stress, poor physiological condition, and/or reduced growth rates—whether linked to fishing pressure or other environmental factors—could theoretically have increased TEFs, resulting in rising *δ*^15^N values without underlying trophic level changes; and (ii) overfishing of cod and herring from the nineteenth century may have disrupted trophic dynamics sufficiently to result in measurable changes in the trophic level of cod of a given size, perhaps in part owing to the impact on age/size structure of the cod themselves. Given the complexity of marine food webs, ecological modelling with a wider set of input variables would be required to explore this possibility further.

## Data Availability

The datasets and computer codes for this article are archived at the Zenodo digital repository [81]. Supplementary material is available online [82].
